# Biological and Chemical Diversity of Bacteria Associated with a Marine Flatworm

**DOI:** 10.3390/md15090281

**Published:** 2017-09-01

**Authors:** Hui-Na Lin, Kai-Ling Wang, Ze-Hong Wu, Ren-Mao Tian, Guo-Zhu Liu, Ying Xu

**Affiliations:** 1 Shenzhen Key Laboratory of Marine Bioresource & Eco-Environmental Science, Shenzhen Engineering Laboratory for Marine Algal Biotechnology, College of Life Sciences and Oceanography, Shenzhen University, Shenzhen 518060, China; timaroona@163.com (H.-N.L.); kailingw@163.com (K.-L.W.); 2 School of Life Sciences, Xiamen University, Xiamen 361102, China; 3 Key Laboratory of Marine Drugs, Ministry of Education of China, School of Medicine and Pharmacy, Ocean University of China, Qingdao 266003, China; 4 The Eighth Affiliated Hospital, Sun Yat-sen University, Shenzhen 518033, China; wuzehong922@126.com; 5 Integrated Chinese and Western Medicine Postdoctoral Research Station, Jinan University, Guangzhou 510632, China; 6 Division of Life Science, The Hong Kong University of Science and Technology, Clear Water Bay, Kowloon, Hong Kong SAR, China; tianrenmao@gmail.com; 7 HEC Research and Development Center, HEC Pharm Group, Dongguan 523871, China; liuguozhu@hecpharm.com

**Keywords:** marine flatworm, natural products, bacteria, *Paraplanocera* sp., *Streptomyces* sp., geldanamycin

## Abstract

The aim of this research is to explore the biological and chemical diversity of bacteria associated with a marine flatworm *Paraplanocera* sp., and to discover the bioactive metabolites from culturable strains. A total of 141 strains of bacteria including 45 strains of actinomycetes and 96 strains of other bacteria were isolated, identified and fermented on a small scale. Bioactive screening (antibacterial and cytotoxic activities) and chemical screening (ultra-performance liquid chromatography–mass spectrometry (UPLC-MS)) yielded several target bacterial strains. Among these strains, the ethyl acetate (EA) crude extract of *Streptomyces* sp. XY-FW47 fermentation broth showed strong antibacterial activity against methicillin-resistant *Staphylococcus aureus* ATCC43300 (MRSA ATCC43300) and potent cytotoxic effects on HeLa cells. The UPLC-MS spectral analysis of the crude extract indicated that the strain XY-FW47 could produce a series of geldanamycins (GMs). One new geldanamycin (GM) analog, 4,5-dihydro-17-*O*-demethylgeldanamycin (**1**), and three known GMs (**2**–**4**) were obtained. All of these compounds were tested for antibacterial, cytotoxic, and antifungal activities, yet only GM (**3**) showed potent cytotoxic (HeLa cells, EC_50_ = 1.12 μg/mL) and antifungal (*Setosphaeria turcica* MIC = 2.40 μg/mL) activities. Their structure–activity relationship (SAR) was also preliminarily discussed in this study.

## 1. Introduction

Flatworms are invertebrates that belong to Platyhelminthes. About 4500 species of flatworms have been reported, of which over 1000 species are exclusively marine flatworms primarily belonging to *Turbellaria* polyclad [1]. Marine flatworms are 3–20 cm in length but some are quite small. Most marine flatworms are free-living organisms and they are found to hide under rocks or inside empty shells in the daytime. In recent years, flatworms have attracted broad research interests worldwide. Several studies on the reproductive system of flatworms provided useful information for research on stem cells [2], ageing [3] and bioadhesion [4]. Recent reports showed that flatworms are suitable models for probing environmental changes, such as salinity stress [5] and oxygen concentrations variations [6].

Chemical defense is popular among marine invertebrates [7], such as sponges, corals and sea hares. In many cases, chemical weapons are produced by microorganisms associated with their invertebrate hosts [8,9,10]. Marine flatworms may also adopt chemical defense to protect themselves. Indeed, the deadly toxin tetrodotoxin (TTX) has been detected in marine flatworm *Planocera* sp. [11] and further investigation of *Planocera multitentaculata* indicated that a high concentration of TTX existed in the genitalia, digestive organs and other tissues [12]. There is doubt that TTX and its analogs might be produced by the endogenous/symbiotic bacteria of these flatworms [13]. Unfortunately, only a handful studies [14,15,16,17] have been carried out to investigate the microorganisms associated with flatworms. Gruber-Vodicka et al. [14] discovered the symbiotic relationship between *Paracatenula galateia* and an uncultured sulfur-oxidizing *Alphaproteobacteria* strain. Xu et al. [15], Zhang et al. [16] and Lin et al. [17] described the identification of new bacteria species isolated from a marine flatworm *Paraplanocera* sp. Since Acoelomorpha flatworms had been excluded from Platyhelminthes to be an independent phylum [18], the symbiotic relationship and chemical defense between algae and Acoelomorpha flatworms will not be discussed in this study. Therefore, it will be of interest to investigate the bacteria associated with marine flatworms, which may provide more clues to understand their chemical defensive system. Geldanamycin (GM), firstly reported by Deboer et al. [19], was discovered from *Streptomyces hygroscopicus.* This compound belongs to the benzoquinone ansamycin family possessing a 19-membered macrocyclic lactam. The biosynthesis of GM and its analogs requires polyketide synthase (PKS) genes. GM is an antibiotic with potent anticancer, antibacterial, anti-protozoa and antifungal activities. However, its hepatotoxicity and poor water solubility limit the therapeutic efficiency of GM. Therefore, studies on GM analogs have now gained increasing attention. Accordingly, various GM analogs have been developed by chemical modification and by engineered biosynthesis [20]. Especially, GM and its two semi-synthetic analogs 17-allylaminogeldanamycin (17-AAG) and 17-dimethylaminoethylamino-17-demethoxygeldanamyein (17-DMAG) show a broad spectrum of anticancer activity by inhibiting Hsp90 (Heat shock protein 90) activity [21,22]. Although both 17-AAG and 17-DMAG were used in clinical trials, they failed in phase II and I clinical trials, due to their severe hepatotoxicity, low aqueous solubility and limited oral bioavailability [23]. By now, only a few of natural GM analogs have been discovered from *Streptomyces hygroscopicus* and other *Streptomyces* sp. [24,25,26]. Hence, discovering natural and potent geldanamycins (GMs) with excellent bioactivities without causing the above common problems will be meaningful for the development of anticancer candidates.

**Figure 1:**
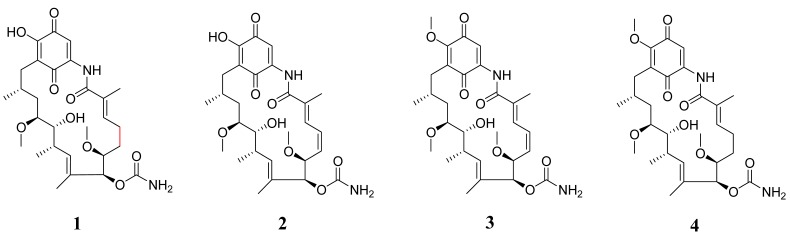
Chemical structures of geldanamycin and its analogs.

In this study, we isolated and identified the bacteria from a marine flatworm *Paraplanocera* sp., conducted bioactive (activity against Methicillin-Resistant *Staphylococcus aureus* (MRSA) ATCC43300 and cytotoxic effects on HeLa cells) and chemical (ultra-performance liquid chromatography–mass spectrometry (UPLC-MS)) diversity screening with the ethyl acetate (EA) crude extracts of the cultured bacteria, and performed further isolation, structure elucidation, and bioactive evaluation for the obtained metabolites of the target strains. One novel GM analog 4,5-dihydro-17-*O*-demethylgeldanamycin (**1**) and three known GMs (**2**–**4**) were isolated from the fermentation broth of XY-FW47 (see Figure 1). All of these compounds were tested for anti-MRSA ATCC43300, cytotoxic, and antifungal activities, and their structure–activity relationship (SAR) was also preliminarily discussed. This is the first report on studying biodiversity and chemical diversity of bacteria associated with marine flatworms, which calls more attention to exploit the biological and chemical potential of marine flatworms.

## 2. Results and Discussion

### 2.1. Sample Identification

**Figure 2:**
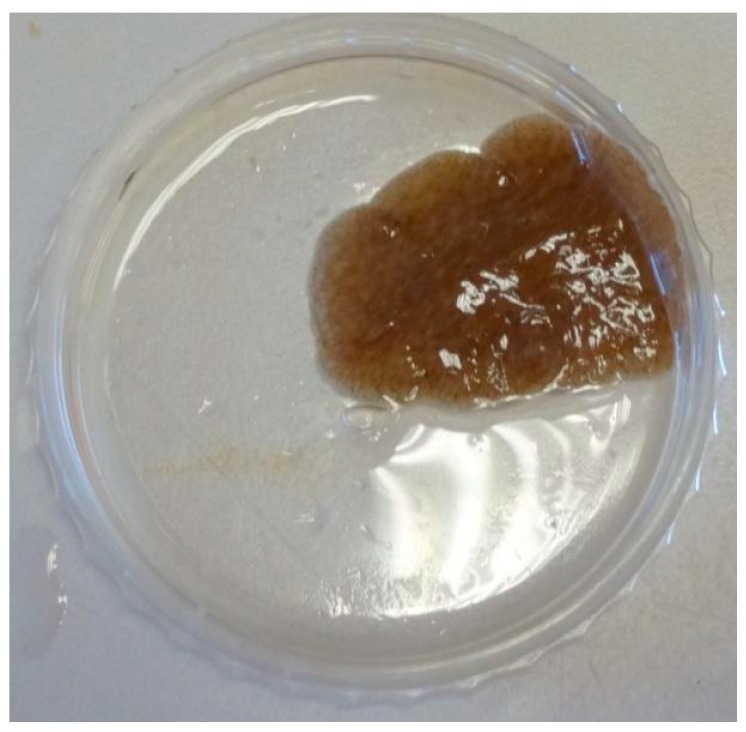
Photo of the *Paraplanocera* sp. used in this study.

**Figure 3:**
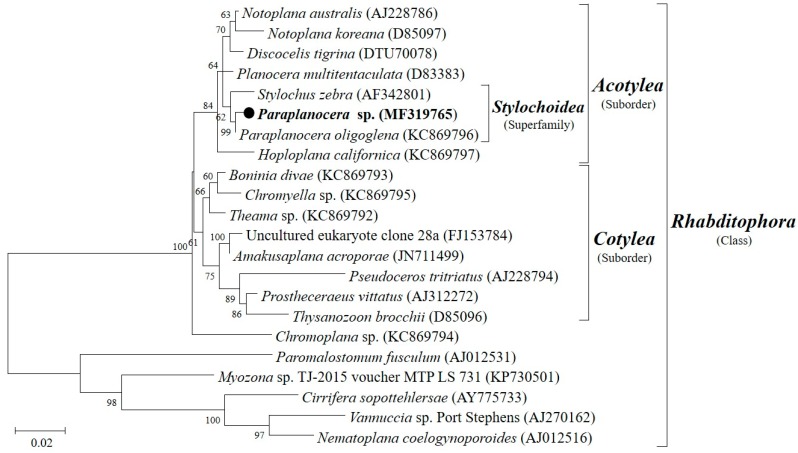
Maximum likelihood tree showing the phylogenetic position of the *Paraplanocera* sp. based on the 18S rRNA gene (1260 bp).

A marine flatworm (Figure 2), collected at depth between 1 and 3 m in the intertidal zone of Yung Shue O, Hong Kong (114°21′ E, 22°24′ N) in January 2014, was originally identified as *Stylochus* sp. by a simple morphological comparison, but was later identified to be *Paraplanocera* sp. based on its 18S rRNA gene sequence (1260 bp). Maximum likelihood tree (Figure 3) shows the phylogenetic position of the *Paraplanocera* sp. which is affiliated to the superfamily of Stylochoidea and forms the closest genetic distance with *Paraplanocera oligoglena*. The GenBank accession number for the 18S rRNA gene sequence of *Paraplanocera* sp. is MF319765.

### 2.2. Isolation and Taxonomy of Bacteria from the Marine Flatworm Paraplanocera *sp.*

A total of 141 strains of bacteria associated with the marine flatworm *Paraplanocera* sp. were isolated and identified, including 37 species of actinobacteria belonging to nine genera and 64 species of non-actinobacteria affiliating to 27 genera (the information of the isolates are detailed in Table 1). The 16S rRNA genes of all the cultured isolates have high similarity (98.5–100%) with their reference strains except the three novel species (marked in bold in Table 1). There were 45 strains of actinomycetes listed as follows, *Streptomyces* (14 strains of 13 species), *Micromonospora* (12 strains of eight species), *Mycobacterium* (nine strains of eight species), *Tsukamurella* (two strains of two species), *Microbacterium* (two strains of two species), *Micrococcus* (two strains of one species), *Pseudonocardia* (two strains of one species), *Brevibacterium* and *Arthrobacter*. There were 96 strains of non-actinomycetes, including *Bacillus* (33 strains of 22 species), *Vibrio* (11 strains of six species), *Halobacillus* (six strains of five species), *Microbulbifer* (four strains of three species), *Ruegeria* (11 strains of two species), *Pseudovibrio* (five strains of three species), *Fictibacillus* (two strains of two species), *Pseudoalteromonas* (two strains of one species), *Photobacterium*, *Joostella* (two strains of one species), *Flammeovirga* (two strains of one species), *Arcobacter*, *Staphylococcus*, *Aquimarina*, *Tenacibaculum*, *Roseovarius*, *Cupriavidus*, *Oceanobacillus*, *Deinococcus*, *Pseudomonas*, *Paenibacillus*, *Stenotrophomonas*, *Paracoccus*, *Psychrobacillus*, *Alcaligenes* (two strains of two species), *Roseivirga* and *Methylobacterium*. These results showed a great diversity of culturable bacteria associated with marine flatworm *Paraplanocera* sp.

Three novel bacterial strains were discovered from this study. Two strains, UST20140214-052 and UST20140214-015B, had been characterized to be new species of the genus *Pseudovibrio* and published elsewhere [15,16]. In these years, there is a growing interest in *Pseudovibrio* species as more and more isolates have been identified from sponges, corals, and sea squirts and, among them, some strains have genomic interactions with host marine invertebrates and gene clusters for producing secondary metabolites to protect the host from pathogens [27]. Another new species strain, XY-FW106, was characterized to be *Deinococcus planocerae* [17]. Interestingly, XY-FW106 showed resistance against ultraviolet irradiation [28], which might provide some protective function for the survival of the host flatworm, which lived in the shallow waters.

### 2.3. The Bioactive and UPLC-MS Chemical Screening of the Isolated Bacteria

All 141 bacterial isolates were fermented on a small scale and the EA crude extracts of their bacterial fermentation broth were evaluated for anti-MRSA (strain ATCC43300) and cytotoxic (HeLa cells) activities. In total, eight extracts showed anti-MRSA activity and seven showed cytotoxic activity. Among them, extracts of strains XY-FW47 and XY-FW120 showed both potent antibacterial and cytotoxic activities. Four strains of *Streptomyces* showed the strongest anti-MRSA activity. In addition, one *Arthrobacter soli* strain and one *Bacillus siamensis* strain showed moderate activity against MRSA, and the latter strain also showed weak effects on HeLa cells (Table 2 and Table 3). Two strains of *Streptomyces* revealed high activity against HeLa cells, while two other *Streptomyces* strains and one *Paracoccus* strain showed moderate activity against HeLa cells (Table 3). These results suggested that the bacteria associated with the marine flatworms might be a rich source of bioactive compounds. In this study, the strains of XY-FW47 and XY-FW120 were selected as the target strains for further chemical characterization due to their strongest bioactivities.

**Figure 4:**
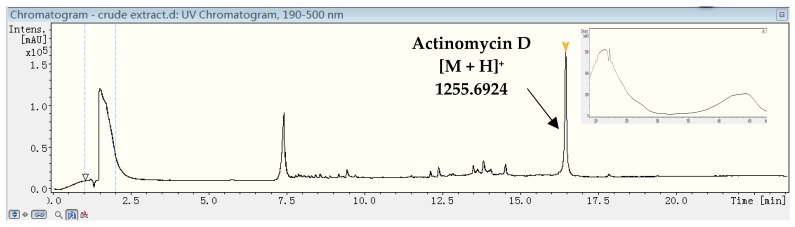
Ultra-performance liquid chromatography–mass spectrometry (UPLC-MS) analysis and ultraviolet (UV) spectra of the crude extract of XY-FW120. The main metabolite produced by XY-FW120 is actinomycin D.

Further chemical analyses on the two strains of *Streptomyces* (XY-FW120 and XY-FW47) were proceeded by UPLC-MS on a reversed phase C18 column with a gradient solution ACN/H_2_O (5–95%, 25 min). As shown in Figure 4, XY-FW120 produced a main metabolite with a characteristic ultraviolet (UV) absorption of 430–440 nm and a high resolution electrospray ionization mass spectroscopy (HR-ESI-MS) [M + H]^+^ of 1255.6924. Combined database searching (Dictionary of natural products, SciFinder, AntiBase, MarinLit, etc.) with extensive literature searching suggested this metabolite was very likely to be actinomycin D (see Figure 4 and Figure S9) which had already been described by Meienhofer and Atherton [29]. As actinomycin D is a well-known antibiotic with antitumor activity, this also explains why the extract of XY-FW120 showed strong activity in both bioassays. It was also reported that the same species *Streptomyces parvulus* DAUFPE 3124 produced only actinomycin D [30]. However, the strain XY-FW120 produced not only a high level of actinomycin D, but also a few known actinomycin D analogs with trace amounts (data not shown). As the UV absorption patterns as well as the high resolution mass data of these actinomycin compounds were the same as those described in the literature, there is a very high chance that these compounds are exactly the same as those reported. However, there might be one case that our consumption could be wrong: enantiomers are also possible, although the odds are low. Based on these analyses, the metabolites of strain XY-FW120 are almost fully understood, thus we did not proceed for further investigation on this strain to characterize its metabolites.

**Figure 5:**
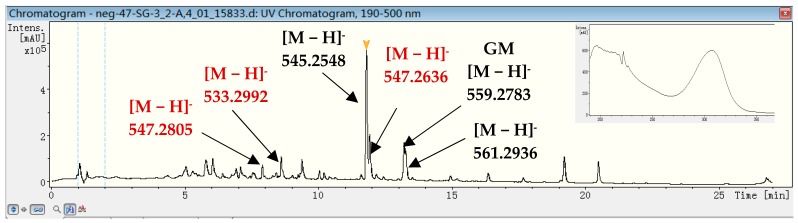
UPLC-MS analysis and UV spectra of the crude extract of XY-FW47: (1) compounds with HR-ESI-MS [M − H]^−^ 547.2805, 533.2992 and 547.2636 in red are very likely novel GMs (highlighted by red color); and (2) compounds with HR-ESI-MS [M − H]^−^ 545.2548, 559.2783 and 561.2936 in black show to be known 17-*O*-demethylgeldanamycin, GM and 4,5-dihydrogeldanamycin (highlighted by black color).

HPLC analysis of the metabolites of XY-FW47, which was closely related to *Streptomyces samsunensis* M1463^T^ [31], indicated a series of compounds with very similar UV absorption patterns. All of these compounds all had characteristic UV absorption at 305 nm with their HR-ESI-MS [M − H]^−^ ranging from 533.2987 to 561.2936 (see Figure 5). Combined database searching with extensive literature searching based on these spectroscopic data, led us to conclude that these compounds with HR-ESI-MS [M − H]^−^ 559.2778, 545.2548 and 561.2936 were GM, 17-*O*-demethylgeldanamycin and 4,5-dihydrogeldanamycin, respectively [19,32,33]. GM, previously isolated from *Streptomyces hygroscopicus*, is a benzoquinone antibiotic containing the typical structures of benzoquinone and dienamide. This class of compounds showed various bioactivities, including anticancer, anti-protozoa, antimalarial and antifungal activities [34,35]. Based on extensive investigation of the literatures and the analyses of UPLC-MS data, it is concluded that these compounds with HR-ESI-MS [M − H]^−^ 547.2805 (retention time at 8.0 min), 533.2992 and 547.2636 (retention time at 12.0 min) are very likely novel GMs produced by the strain XY-FW47 (see Figure S10). As new analogs may provide more selectivity or low toxicity, it is worth isolating these potential new GMs. Consequently, this bacterial strain was chosen to be the target strain for further study. The 16S rRNA gene sequence of strain XY-FW47 was deposited in GenBank with the accession number MF664376.

### 2.4. Structural Elucidation of the Isolated Compounds

In total, four compounds were isolated and identified from strain XY-FW47. Compound **1** was isolated as a yellow amorphous powder. The molecular formula of Compound **1** was established as C_28_H_40_N_2_O_9_ by HR-ESI-MS (*m*/*z* 547.2636, [M − H]^−^), with 10 degrees of unsaturation. The ^13^C and DEPT NMR spectra showed 28 carbon signals (Table 4), which were assigned with the assistance of the distortionless enhancement by polarization transfer (DEPT) spectrum to six methyls, four methylenes, seven methines, and nine quaternary carbons. The signals of ^1^H-NMR spectra also revealed six methyl groups (*δ*_H_ 3.41, 3.35, 1.91, 1.68, 0.98, and 0.96) and four methines.

**Figure 6:**
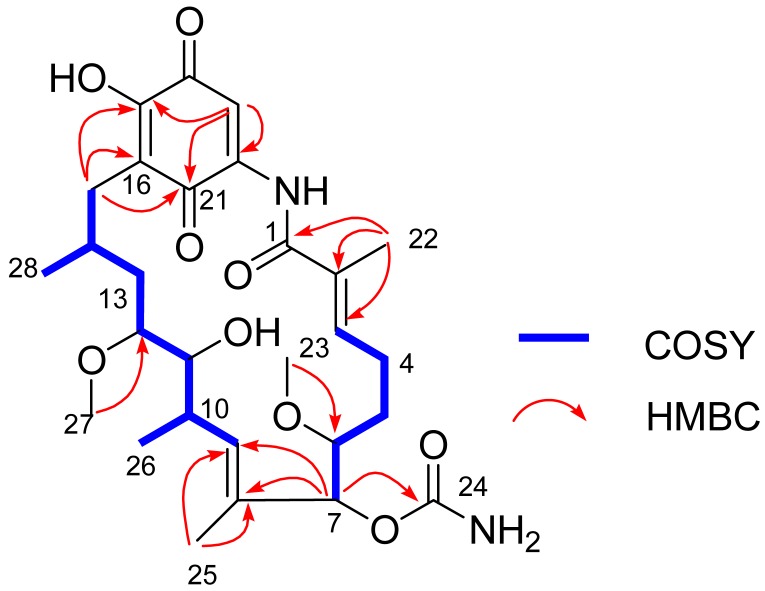
Key COSY and HMBC correlations of Compound **1**.

The gross structure of Compound **1** and all of the ^1^H and ^13^C NMR data associated with the molecule were determined by 2D NMR studies, including ^1^H-^1^H COSY, HSQC and HMBC experiments. The key ^1^H-^1^H COSY correlations of H-3/H_2_-4/H_2_-5/H-6/H-7, H-9/H-10/H-11/H-12/H_2_-13/H-14/H_2_-15, H-10/CH_3_-26 and H-14/CH_3_-28, together with the HMBC correlations from CH_3_-22 to C-1/C-2/C-3, from OCH_3_-23 to C-6, from H-7 to C-8/C-9/C-24, from CH_3_-25 to C-8/C-9 and from CH_3_-27 to C-12, indicated the existence of the ansa ring (see Figure 6), which was similar to 17-*O*-demethylgeldanamycin [32]. Comparing with the 1D and 2D NMR signals of 17-*O*-demethylgeldanamycin, the lost of the double bond at C-4 and C-5 in Compound **1**, which was replaced by two methenes. The NOSY correlations of H-6/H-7/H-12, and H-10/H-11/H-14 indicated the absolute configuration of Compound **1** that was also consistent with 17-*O*-demethylgeldanamycin. Thus, the structure of Compound **1** was identified.

The structures of Compounds **2**–**4** were identified as 17-*O*-demethylgeldanamycin, geldanamycin and 4,5-dihydrogeldanamycin, respectively, by the NMR and UPLC-MS data, and comparing their spectroscopic data with those data reported by Hong et al. and Ni et al. [36,37].

### 2.5. Bioactive Evaluation and Structure–Activity Relationship

The bioactivities (anti-MRSA, anti-HeLa Cell and antifungal) of Compounds **1**–**4** were evaluated. These compounds showed no activity against MRSA. Compound **3** GM exhibited potent activity against HeLa cells with EC_50_ 1.12 μg/mL. It also showed antifungal activity against the plant pathogen *Setosphaeria turcica* with MIC 2.40 μg/mL. Previous cytotoxic tests (FRE/*erbB-2* tumors) of 4,5-dihydrogeldanamycin (**4**) revealed much weaker activity than GM (IC_50_ = 230 and 70 nM, respectively) [38]. Compounds 17-*O*-demethylgeldanamycin (**2**) and 17-*O*-demethylgeldanamycin hydroquinone showed much more cytotoxicity towards normal P19-derived neurons than GM at 1 nM [39]. These data together with the present results of Compound **1**–**4** indicate that the double bond at C-4,5 and the methoxy group at C-17 position are essential for increasing anti-HeLa cells and anti-*S. turcica* activities of GMs. As none of the GMs were active against MRSA, the potent anti-MRSA activity of the crude XY-FW47 extract might be contributed by the trace GMs or other types of metabolites which we were not able to isolate. We will need to optimize fermentation conditions of XY-FW47 to obtain enough quantities of these compounds in the future studies.

## 3. Materials and Methods

### 3.1. Bacteria Isolation and Identification

A marine flatworm was collected at depth between 1 and 3 m intertidal zone of Yung Shue O, Hong Kong (114°21′ E, 22°24′ N) in January 2014, and identified as *Paraplanocera* sp. based on 18S rRNA gene sequencing (1260 bp). The bacterial isolation method was in accordance with the method described by Xu et al. [15] using modified BD Difco^TM^ R2A agar (adding 17 g/L seasalt). The 16S rRNA gene sequence of the isolations were determined by PCR using universal primers 27F and 1492R, then the isolates were identified by the blast program in the NCBI database.

### 3.2. Bacteria Fermentation

Small scale fermentation of the cultured bacteria was carried out as follows: seed cultures of the strain were collected in 50 mL Falcon centrifuge tubes, with each containing 15 mL of SGTYP medium with sea salts (5.0 g soluble starch, 5.0 g glucose, 1.0 g tryptone, 1.0 g yeast extract, 1.0 g peptone, 17.0 g sea salts per litre, pH 7.6 ± 0.2). Then fresh inoculum was inoculated in 250 mL flasks with each containing 80 mL of SGTYP medium with sea salts (5.0 g soluble starch, 5.0 g glucose, 1.0 g tryptone, 1.0 g yeast extract, 1.0 g peptone, 17.0 g sea salts per litre, pH 7.6 ± 0.2). The flasks were incubated at 28 °C for 5 days before harvesting. The fermentation broth was extracted with ethyl acetate (EA) three times of the total volume (1:3 *v*/*v*). The EA crude extract of the bacterial fermentation broth was obtained and prepared as 50 mg/mL stock solution in DMSO for testing.

### 3.3. Bioactive and Chemical Screening

The pathogen MRSA ATCC43300 was incubated in LB broth (10 g tryptone, 5 g yeast extract and 10 g NaCl per liter) at 28 °C for 12 h and then diluted 5000 times with fresh LB broth. The tested samples (2 μL) were added to each well of 24-well plates with 1 mL of the diluted pathogen solution. The pathogen was then incubated at 28 °C and the optical density at 600 nm was measured 24 h after inoculation with vancomycin (50 μg/mL) as positive controls. The cytotoxic assays were performed using the method described by Li et al. [40]. HeLa cells were inoculated and incubated in 24-well plates for 12 h before adding the tested samples. After incubation for 48 h, the CCK method was used to assay the cell viability. Three biological replicates were carried out for each sample and each bioassay experiment was repeated three times.

The ESI-TOF and mass spectra of the isolates were acquired from a UPLC-TOF-MS system (ultra-performance liquid chromatography–time of fly-mass spectrometry) using a Bruker microTOF-q II (Bruker Daltonics GmbH, Bremen, Germany) mass spectrometer coupled to a Waters ACQUITY UPLC system (Waters, London, UK).

### 3.4. Extraction and Compounds Isolation

Large scale fermentation (50 L) and the extraction of XY-FW47 were obtained as the described method of small scale. The EA crude extract of XY-FW47 was separated by reverse phase C18 chromatography with water and methanol solvent mixtures of H_2_O–MeOH (7:3), H_2_O–MeOH (5:5), H_2_O–MeOH (3:7), H_2_O–MeOH (1:9), and 100% MeOH. The fractions of H_2_O–MeOH (3:7, *v*/*v*) was evaporated and labeled Fraction (Fr.) 70%. The fraction was subjected to Sephadex LH-20 by mixtures of chloroform/methanol (1:1) yielding 16 fractions and marked Fr.70%-1 to Fr.70%-16. Then, Fr.70%-10 to Fr.70%-14 were purified by the elution of MeCN–H_2_O (70:30, (*v*/*v*), flow rate: 2 mL/min) through the semi-preparative HPLC (Waters, Parsippany, NJ, USA) using analytical and semi-preparative reverse-phase phenomenex biphenyl columns (5 μm, 250 × 4.6 mm and 5 μm, 250 × 10 mm in size), and finally afforded pure Compounds **1** (2.6 mg), **2** (12 mg), **3** (7 mg) and **4** (1 mg) at a retention time of 24.0 min, 25.0 min, 26.0 min and 27.4 min, respectively. The further ^1^H, ^13^C and 2D NMR spectral data were determined on a Bruker DRX 600 MHz NMR Spectrometers.

4,5-Dihydro-17-*O*-demethylgeldanamycin (**1**): yellow amorphous powder; ${\left[\alpha \right]}_{D}^{25}$ +42.0° (*c* 0.18, CHCl_3_); UV (MeOH) λ_max_ 305 nm, HR-ESI-MS *m*/*z* 547.2636 [M − H]^−^ (calcs for C_28_H_40_N_2_O_9_ 547.2656); ^1^H and ^13^C NMR data, see Table 4.

### 3.5. Bioactive Assays

The tests of Compounds **1**–**4** against MRSA ATCC43300 were determined as previously described. The antifungal activities against three plant pathogens (*Setosphaeria turcica*, *Bipolaris maydis* and *Altemaria solani*) were conducted on BD Difco^TM^ Potato Dextrose Agar at 28 °C for 24 h. A series of two-fold dilution of tested samples was made with either LB broth or Potato Dextrose Broth in 24-well plates. The antibacterial tests were checked as the method described in the Section 3.3. The antifungal tests were performed at 28 °C for 24 h, and inhibition of ≥95% of the growth was observed by stereo microscope.

The cytotoxic tests were measured by using the previous method. Compounds **1**–**4** were prepared as 50 mg/mL in DMSO, and a series of two-fold dilution was made with the assayed media. After 48 h of incubation, the cytotoxicities were assayed by the CCK method.

## 4. Conclusions

This study firstly explored biological and chemical diversity of bacteria associated with the marine flatworm *Paraplanocera* sp. A total of 141 strains of bacteria were isolated including 45 strains of actinomycetes and 96 strains of other bacteria. Among them, there were three novel strains, suggesting that a rich biodiversity of bacteria may be associated with marine flatworms. The isolation and identification results of these bacteria indicated biological diversity and novelty of bacteria derived from this marine flatworm. One new GM analog (**1**) and three known GMs (**2**–**4**) were obtained from *Streptomyces* sp. XY-FW47. GM (**3**) showed potent bioactivity against HeLa cells with EC_50_ 1.12 μg/mL and against plant fungal pathogen *Setosphaeria turcica* with MIC 2.40 μg/mL. Preliminary discussion of SAR suggested that the existence of C-17 methoxy group and C-4,5 double bond might increase the bioactivities of GMs. Our study has provided new insights into the bacteria associated with marine flatworms.

## Figures and Tables

**Table 1:** The diversity of the culturable bacteria derived from the marine flatworm *Paraplanocera* sp. A total of 141 strains of bacteria, including 37 species of actinobacteria and 64 species of non-actinobacteria, were identified by comparison 16S rDNA sequences of the isolates with their reference strains in the GeneBank of NCBI. All the known isolates have high identity percentage values with 98.5–100% except the three new species (*Pseudovibrio hongkongenesis* UST20140214-015B [15], *Pseudovibrio stylochi* UST20140214-052 [16], and *Deinococcus planocerae* XY-FW106 [17]).

	Species	Isolate ID	Accession Number of the Most Similar Strain	Species	Isolate ID	Accession Number of the Most Similar Strain
Actinobacteria	*Streptomyces libani*	XY-FW38	JN180219	*Mycobacterium pallens*	XY-FW60	KF378757
	*S. rutgersensis*	XY-FW46	FJ99174	*M. parafortuitum*	XY-FW63	NZ_MVID01000062
	*S. samsunensis*	XY-FW47	MF664376	*M. peregrinum*	XY-FW64	AF537362
	*S. sudanensis*	XY-FW121	EF515876	*M. kumamotonense*	XY-FW71	AB239925
	*S. tendae*	XY-FW57W	D63873	*M. poriferae*	XY-FW80	JN627174
	*S. luridus*	XY-FW100W	AB184150	*M. chubuense*	XY-FW83	JYNX01000034
	*S. alboflavus*	XY-FW107	JNXT01000131	*M. bacteremicum*	XY-FW90	FJ172308
	*S. cirratus*	XY-FW109	AY999794	*M. iranicum*	XY-FW62	HQ009482
	*S. parvulus*	XY-FW120	AB184326		XY-FW77	HQ009482
	*S. bacillaris*	XY-FW137	AB184439	*Microbacterium saperdae*	XY-FW102	AB004719
	*S. cavourensis*	XY-FW153	AB184264			
	*S. daliensis*	XY-FW154	AY785161	*M. resistens*	XY-FW145	BCRA01000173
	*S. phytohabitans*	XY-FW134	JQ345722	*Tsukamurella tyrosinosolvens*	XY-FW86	FJ643549
		XY-FW142	JQ345722			
	*Micromonospora eburnea*	XY-FW94	AB107231	*T. strandjordii*	XY-FW101	AF283283
	*M. schwarzwaldensis*	XY-FW95	KC517406	*Brevibacterium iodinum*	XY-FW82	X76567
	*M. carbonacea*	XY-FW99	KM370042	*Micrococcus yunnanensis*	XY-FW48A	FJ214355
	*M. tulbaghiae*	XY-FW123	EU196562		XY-FW75	FJ214355
	*M. echinospora*	XY-FW130	KY818670	*Arthrobacter soli*	XY-FW120A	EF660748
	*M. marina*	XY-FW132	KM370074	*Pseudonocardia carboxydivorans*	XY-FW148	EF114314
	*M. wenchangensis*	XY-FW65	JQ768361			
	*M. aurantiaca*	XY-FW69	CP002162		XY-FW149	EF114314
		XY-FW97	CP002162			
		XY-FW122	CP002162			
Non-actinobacteria	*Bacillus lehensis*	XY-FW24	KX082867	*M. variabilis*	XY-FW48B	KP640585
	*B. pumilus*	XY-FW37	AF492815		XY-FW54	KP640585
	*B. qibsonii*	XY-FW43	KM036072	*Ruegeria arenilitoris*	XY-FW31	JQ807219
	*B. aerius*	XY-FW50	KY243945		XY-FW32	JQ807219
	*B. berkeleyi*	XY-FW53	KR476448		XY-FW40	JQ807219
	*B. algicola*	XY-FW55	KY580789		XY-FW45	JQ807219
	*B. altitudinis*	XY-FW66	ASJC01000029		XY-FW58	JQ807219
	*B. amyloliquefaciens* subsp. *plntruim*	XY-FW105	FN597644		XY-FW72	JQ807219
					XY-FW96	JQ807219
	*B. aerophilus*	XY-FW112	KX951942		XY-FW138	JQ807219
	*B. flecus*	XY-FW125	NZ_JANV01000041	***Pseudovibrio hongkongenesis***	UST20140214-015B	KP207599
	*B. invictus*	XY-FW131	KF060662	[15]		
	*B. megaterium*	XY-FW1A	MF597792	***P. stylochi*** [16]	UST20140214-052	KP207600
	*B. aquimaris*	XY-FW5	MF429570	*P. ascidiaceicola*	XY-FW21B	LN812993
		XY-FW23	KY777466		XY-FW51	LN812993
		XY-FW49	KY753251		XY-FW57	LN812993
	*B. marisflavi*	XY-FW30	MF062965	*Fictibacillus barbaricus*	XY-FW78	KY436215
		XY-F W59	MF062965	*F. phosphorivorans*	XY-FW42	KY471632
	*B. aryabhattai*	XY-FW110	EF114313	*Photobacterium swingsii*	XY-FW3	KY229808
		XY-FW117	EF114313	*Arcobacter nitrofigilis*	XY-FW17	EU106662
	*B. nealsonii*	XY-FW116	EU656111	*Staphylococcus epidermidis*	XY-FW21A	MF429388
		XY-FW126	EU656111	*Aquimarina mueller*	XY-FW21C	AY608408
		XY-FW133	EU656111	*Tenacibaculum aiptasiae*	XY-FW28	KC178948
		XY-FW139	EU656111	*Roseovarius aestuarii*	XY-FW56	EU156066
	*B. idriensis*	XY-FW113	AY904033	*Cupriavidus campinensis*	XY-FW67	KY010351
		XY-FW115	AY904033	*Oceanobacillus picturae*	XY-FW73	KX068643
		XY-FW118	AY904033	***Deinococcus planocerae*** [17]	XY-FW106	KT886059
		XY-FW129	AY904033	*Pseudomonas libanensis*	XY-FW111	KY933473
		XY-FW136	AY904033	*Paenibacillus cineris*	XY-FW114	AJ575658
	*Vibrio cyclitrophicus*	XY-FW7B	KY382786	*Stenotrophomonas rhizophila*	XY-FW119	CP007597
	*V. chagasii*	XY-FW19	LN832958	*Paracoccus honmiensis*	XY-FW124	DQ342239
	*H. locisalis*	XY-FW34A	JQ799098	*Alcaligenes aquatilis* subsp. *phenolicus*	XY-FW141	JX986974
	*H. halophilus*	XY-FW34B	KX507262		XY-FW146	AUBT01000026
	*H. trueperi*	XY-FW68	LT635432			
	*H. alkaliphilus*	XY-FW10	NZ_FOOG01000089			
		XY-FW16	NZ_FOOG01000089			
	*Microbulbifer elongates*	XY-FW27	KY176867			
	*M. epialgicus*	XY-FW39	KT758460			

**Table 2:** The antibacterial activity against methicillin-resistant *Staphylococcus aureus* ATCC43300 (MRSA ATCC43300) of the bacteria associated with *Paraplanocera* sp.

Bacteria Associated with *Paraplanocera* sp.		Tested Pathogen
Isolate ID	Closest Described Species	MRSA ATCC43300
XY-FW47	*Streptomyces samsunensis*	+++
XY-FW142	*Streptomyces phytohabitans*	+++
XY-FW153	*Streptomyces cavourensis*	+++
XY-FW120	*Streptomyces parvulus*	+++
XY-FW120A	*Arthrobacter soli*	++
XY-FW105	*Bacillus siamensis*	++
XY-FW56	*Roseovarius aestuarii*	+
XY-FW48A	*Micrococcus yunnanensis*	+

Final sample concentration: 100 μg/mL; + represents weak activity (0.2 < OD ≤ 0.4); ++ represents moderate activity (0.1 < OD ≤ 0.2); +++ represents high activity (0.05 < OD ≤ 0.1); Negative control with DMSO (OD ≈ 0.8); Positive control with vancomycin (OD ≈ 0.05).

**Table 3:** The cytotoxic activity of the bacteria associated with *Paraplanocera* sp.

Bacteria Associated with *Paraplanocera* sp.		Cell Line
Isolate ID	Closest Described Species	HeLa Cell
XY-FW47	*Streptomyces samsunensis*	+++
XY-FW120	*Streptomyces parvulus*	+++
XY-FW142	*Streptomyces phytohabitans*	++
XY-FW153	*Streptomyces cavourensis*	++
XY-FW124	*Paracoccus honmiensis*	++
XY-FW105	*Bacillus siamensis*	+
XY-FW100W	*Streptomyces luridus*	+

Final sample concentration: 100 μg/mL; + represents weak activity (0.2 < OD ≤ 0.4); ++ represents moderate activity (0.1 < OD ≤ 0.2); +++ represents high activity (0.05 < OD ≤ 0.1); Negative control with DMSO (OD ≈ 0.8).

**Table 4:** ^1^H and ^13^C NMR data for Compound **1** in CDCl_3_.

Position	*δ*_C_	*δ*_H_ (*J* in Hz)	Position	*δ*_C_	*δ*_H_ (*J* in Hz)
1	168.3, C		16	117.2, C	
2	133.1, C		17	152.8, C	
3	138.4, CH	6.25, t (6.7)	18	183.0, C	
4	24.4, CH_2_	2.42, m	19	107.4, CH	7.28, s
5	29.6, CH_2_	1.77, m	20	140.8, C	
6	80.9, CH	3.37, m	21	184.3, C	
7	80.3, CH	5.18, d (4.2)	NH		8.99, s
8	131.0, C		22-CH_3_	12.3, CH_3_	1.91, s
9	133.8, CH	5.68, d (9.6)	23-OCH_3_	59.0, CH_3_	3.41, s
10	32.5, C	2.77, m	24-OCONH_2_	156.1, C	
11	73.3, CH	3.58, d (7.8)	25-CH_3_	12.5, CH_3_	1.68, s
12	82.5, CH	3.38, m	26-CH_3_	12.7, CH_3_	0.96, d (7.5)
13	34.9, CH_2_	1.73, m	27-OCH_3_	57, CH_3_	3.35, s
14	28.3, CH	1.75, m	28-CH_3_	22.6, CH_3_	0.98, d (7.5)
15	32.0, CH_2_	2.40, m 2.48, dd (13.2, 3.2)			

## Supplementary Materials

The following are available online at www.mdpi.com/1660-3397/15/9/281/S1.
