# Supplementary material for: Biological and Chemical Diversity of Bacteria Associated with a Marine Flatworm
Source: Mar Drugs. 2017 Sep 1;15(9):281. doi: 10.3390/md15090281 (PMC5618420; doi:10.3390/md15090281)

Biological and Chemical Diversity of Bacteria Associated with a Marine Flatworm

Hui-Na Lin[1,2†], Kai-Ling Wang[1,3†], Ze-Hong Wu[4,5], Ren-Mao Tian[6], Guo-Zhu Liu[7],Ying Xu[1*]

[1]Shenzhen Key Laboratory of Marine Bioresource & Eco-environmental Science, Shenzhen Engineering Laboratory for Marine Algal Biotechnology, College of Life Sciences and Oceanography, Shenzhen University, Shenzhen 518060, China; timaroona@163.com (H.-N.L.); kailingw@163.com (K.-L.W.)

[2]School of Life Sciences, Xiamen University, Xiamen 361102, China;

[3] Key Laboratory of Marine Drugs, Ministry of Education of China, School of Medicine and Pharmacy, Ocean University of China, Qingdao 266003, China;

[4]The Eighth Affiliated Hospital, Sun Yat-sen University, Shenzhen, 518033, China; wuzehong922@126.com

[5]Integrated Chinese and Western Medicine Postdoctoral research station, Jinan University, Guangzhou, 510632, China;

[6]Division of Life Science, The Hong Kong University of Science and Technology, Clear Water Bay, Kowloon, Hong Kong SAR, China; tianrenmao@gmail.com

[7]HEC Research and Development Center, HEC Pharm Group, Dongguan 523871, China; liuguozhu@hecpharm.com

† These authors contributed equally to this work.

* Author to whom correspondence should be addressed; E-mail: boxuying@szu.edu.cn; Tel.: +86-755-26958849; Fax: +86-755-26534274.

Supplementary Information

Figure S1. ^{1}H-NMR(600 MHz) of **1** in $CDCl_3$. 2
Figure S2. ^{13}C-NMR(150 MHz) of **1** in $CDCl_3$. 2
Figure S3. DEPT 135 (150 MHz) of **1** in $CDCl_3$. 3
Figure S4. COSY of **1** in $CDCl_3$. 3
Figure S5. HSQC of **1** in $CDCl_3$. 4
Figure S6. HMBC of **1** in $CDCl_3$. 4
Figure S7. NOESY of **1** in $CDCl_3$. 5
Figure S8. High resolution electrospray ionization mass spectroscopy (HR-ESI-MS) spectrum of **1** 5
Figure S9. The chemical structure of actinomycin D 6
Figure S10. The other geldanamycins (GMs) trace of XY-FW47 in ultra-performance liquid chromatography–mass spectrometry (UPLC-MS) (HR-ESI-MS $[M-H]^-$ 547.2805 and 533.2992). 6

Figure S1.^{1}H-NMR(600 MHz) of **1** in $CDCl_3$.

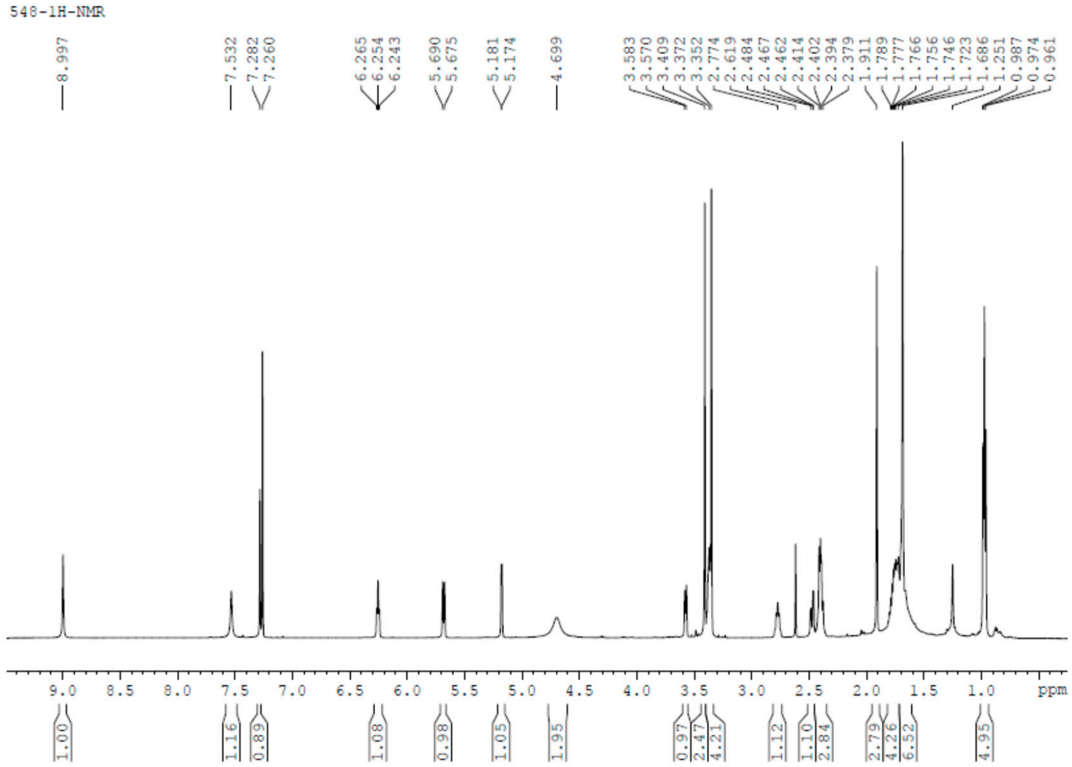

Figure S2. ^{13}C-NMR(150 MHz) of **1** in $CDCl_3$.

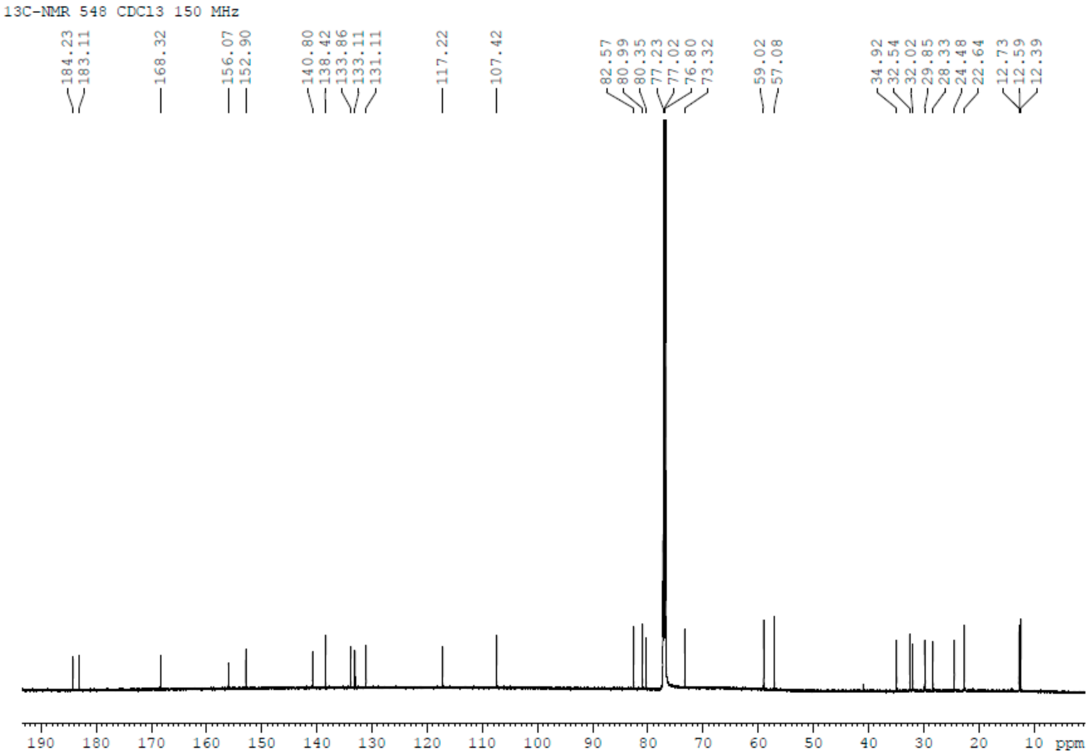

Figure S3. DEPT 135 (150 MHz) of **1** in $CDCl_3$.

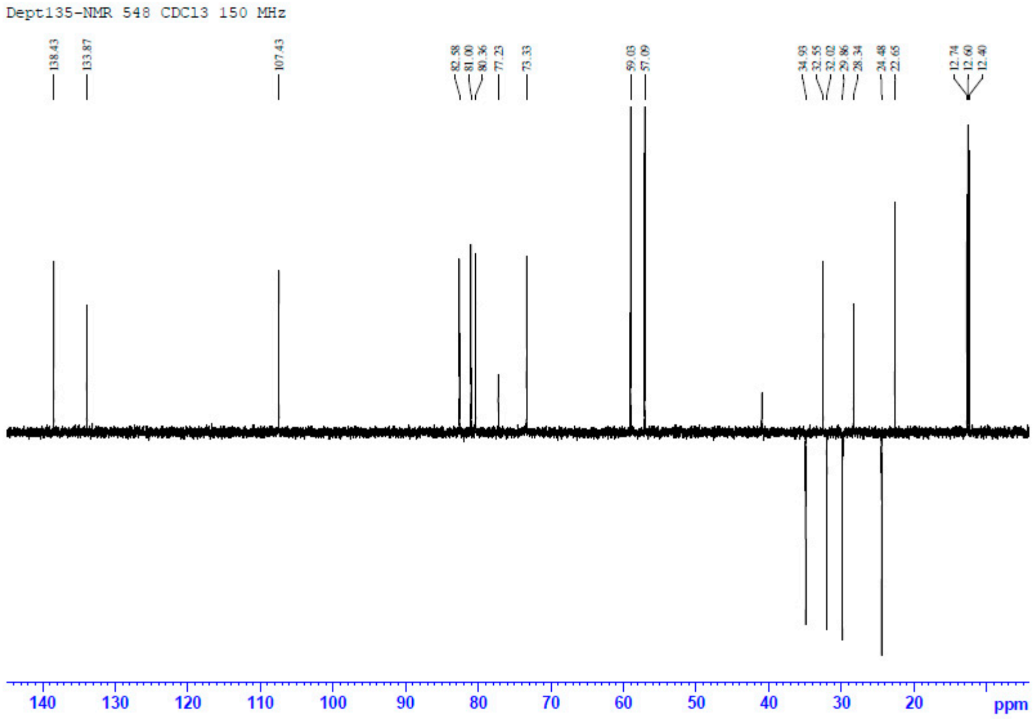

Figure S4. COSY of **1** in $CDCl_3$.

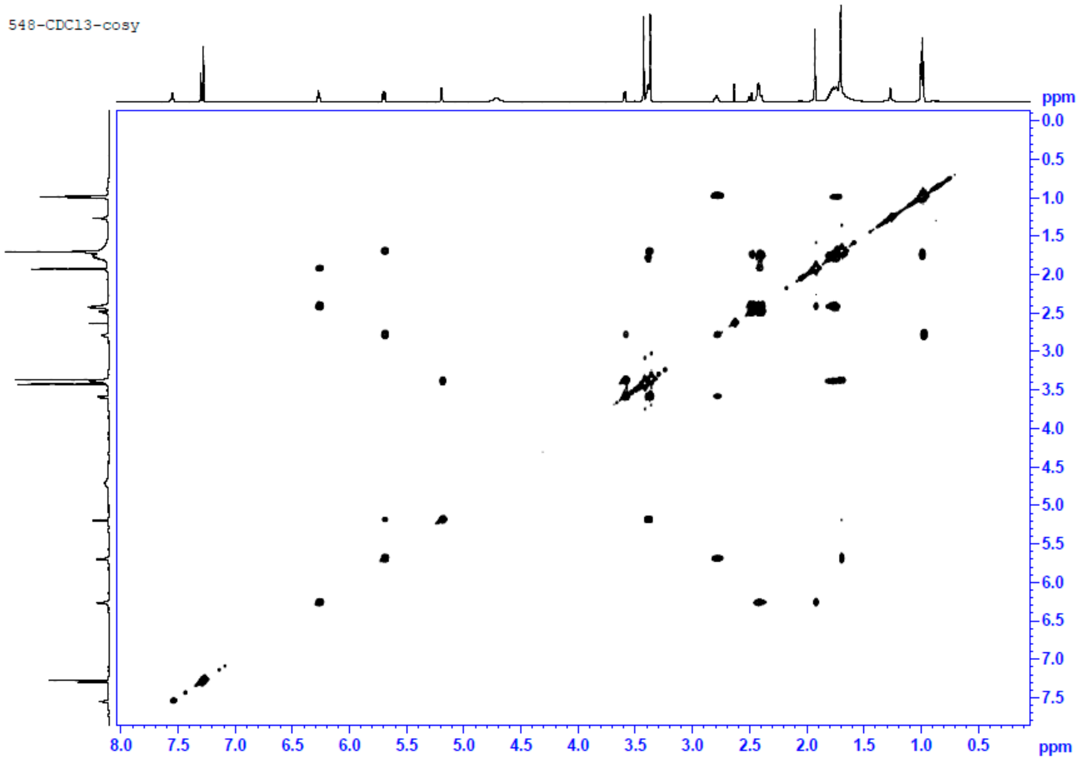

Figure S5. HSQC of **1** in $CDCl_3$.

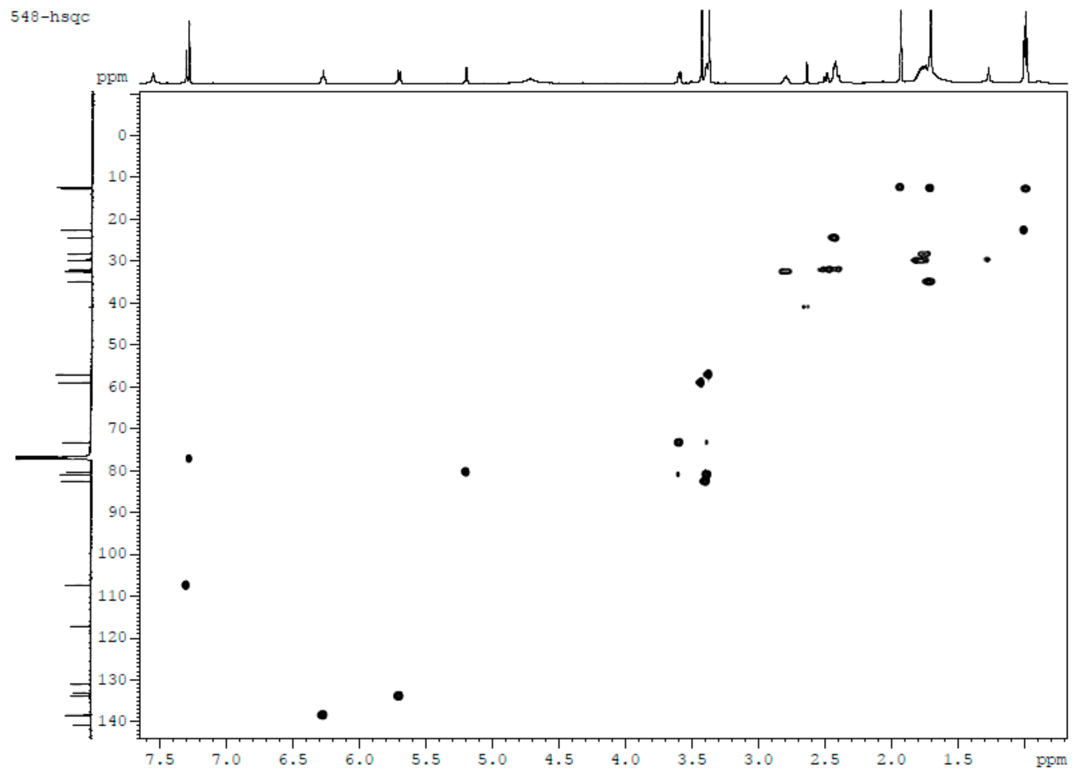

Figure S6. HMBC of **1** in $CDCl_3$.

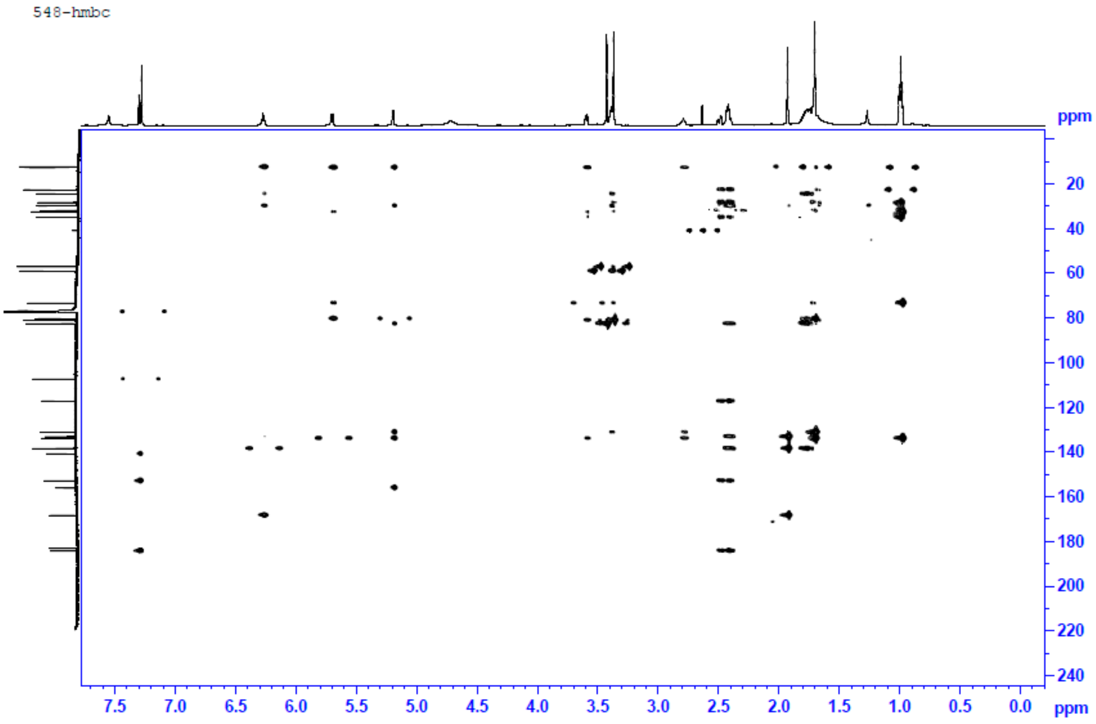

Figure S7. NOESY of **1** in $CDCl_3$.

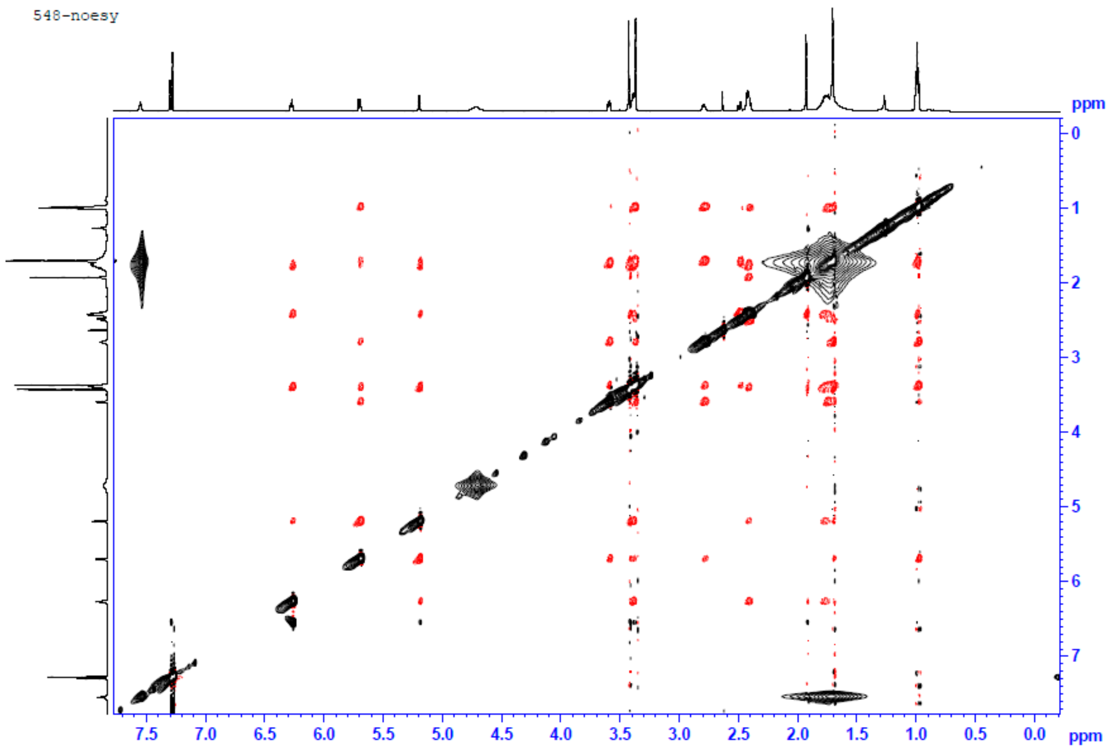

Figure S8. High resolution electrospray ionization mass spectroscopy (HR-ESI-MS) spectrum of **1**.

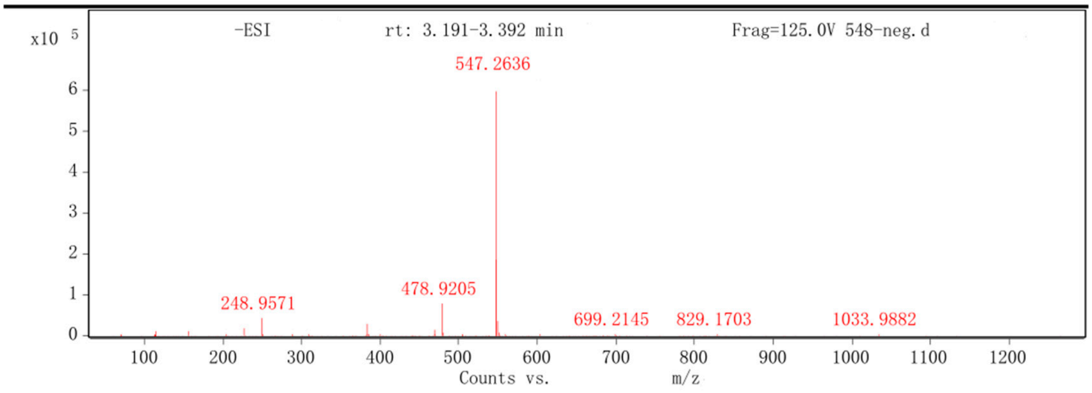

Figure S9. The chemical structure of actinomycin D

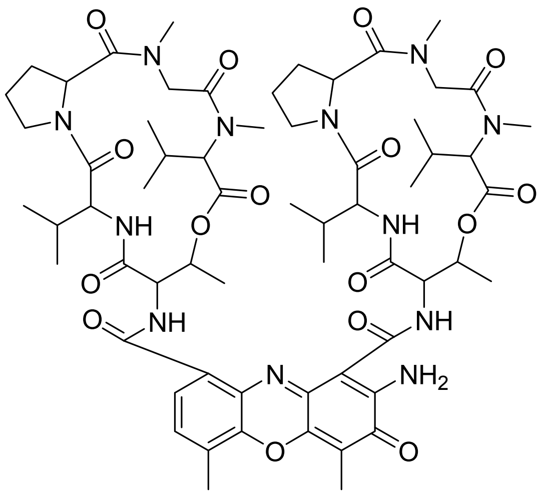

Figure S10. The other geldanamycins (GMs) trace of XY-FW47 in ultra-performance liquid chromatography–mass spectrometry (UPLC-MS) (HR-ESI-MS [M-H]$^-$ 547.2805 and 533.2992).

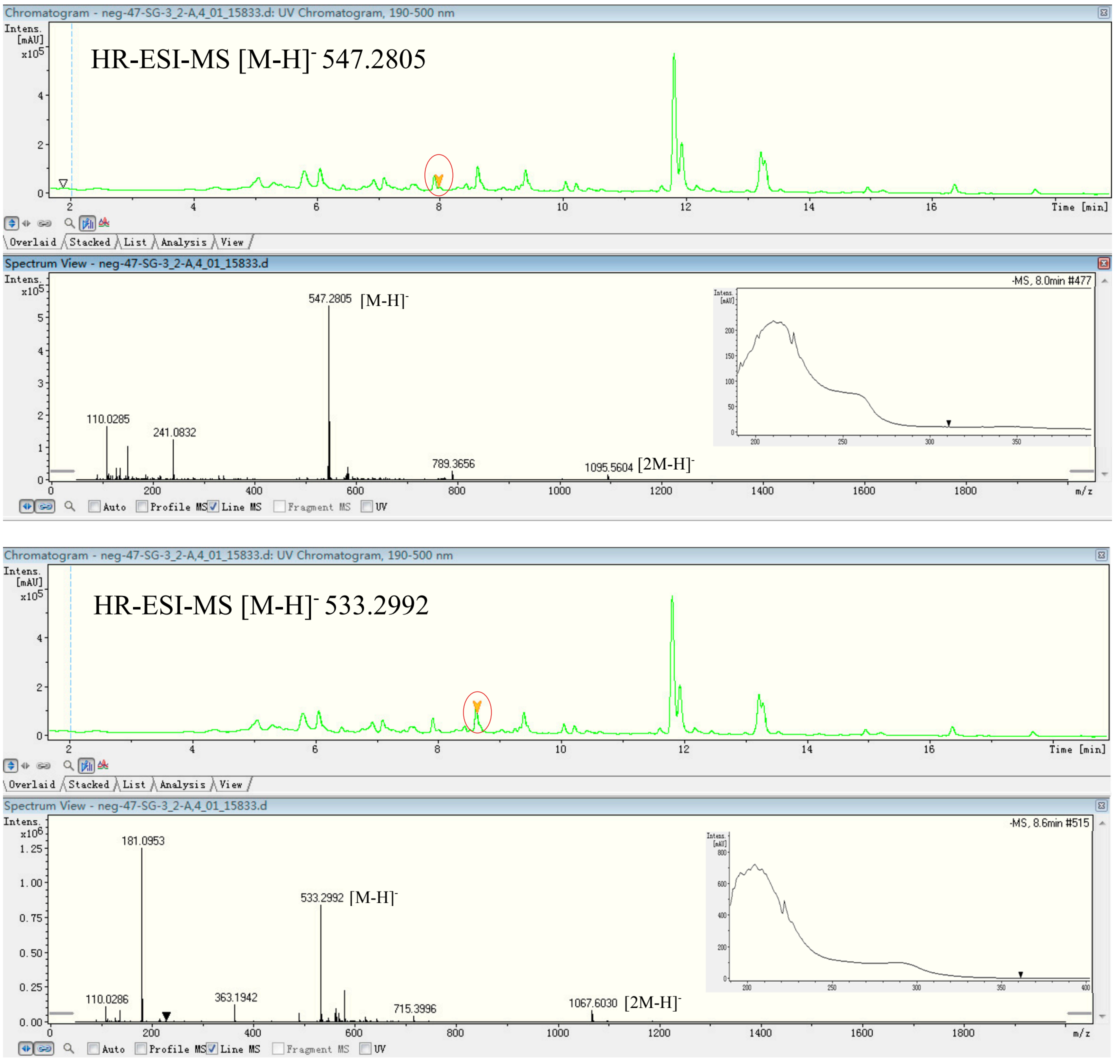

Supplement: Supplementary file 1 [file marinedrugs-15-00281-s001.pdf]
